# Metabolic pathway rewiring in engineered cyanobacteria for solar-to-chemical and solar-to-fuel production from CO_2_

**DOI:** 10.1080/21655979.2017.1317572

**Published:** 2017-05-19

**Authors:** Han Min Woo

**Affiliations:** Department of Food Science and Biotechnology, Sungkyunkwan University (SKKU), Jangan-gu, Suwon, Republic of Korea

**Keywords:** cyanobacteria, metabolic engineering, metabolic pathway rewiring, solar fuel, solar-to-chemical

## Abstract

Photoautotrophic cyanobacteria have been developed to convert CO_2_ to valuable chemicals and fuels as solar-to-chemical (S2C) and solar-to-fuel (S2F) platforms. Here, I describe the rewiring of the metabolic pathways in cyanobacteria to better understand the endogenous carbon flux and to enhance the yield of heterologous products. The plasticity of the cyanobacterial metabolism has been proposed to be advantageous for the development of S2C and S2F processes. The rewiring of the sugar catabolism and of the phosphoketolase pathway in the central cyanobacterial metabolism allowed for an enhancement in the level of target products by redirecting the carbon fluxes. Thus, metabolic pathway rewiring can promote the development of more efficient cyanobacterial cell factories for the generation of feasible S2C and S2F platforms.

## Introduction

Global concerns targeting the reduction of greenhouse gas emissions and sustaining the supply of energy and chemicals have brought attention to the development of sustainable platforms to convert carbon dioxide to chemicals and fuels, in the form of solar-to-chemical (S2C) and solar-to-fuel (S2F) technologies.^1^ The S2C and S2F platforms have been developed to produce the desired value-added chemicals and fuels from 3 elements (CO_2_, H_2_O, and solar energy). Together with the development of integrated bio-electrochemical systems^2,3^ based on engineered lithoautotrophic bacteria for sustainable S2C and S2F production, photoautotrophic cyanobacteria have also been genetically engineered as S2C and S2F platforms to directly produce value-added chemicals from CO_2_. Recent reviews on the development of S2C and S2F platforms using engineered cyanobacteria have focused on general perspectives for cyanobacterial fuels (Cyanofuels),^4^ on the engineering of metabolic pathways in cyanobacteria,^5-7^ on the coupling of enzymes to the photosynthetic reducing power,^8^ and discussed future perspectives from a systems biology point of view.^9^ Thus, I have provided a more detailed analysis of the rewiring of metabolic pathways to increase the carbon flux of CO_2_ toward target end products.

### Metabolic pathway rewiring to improve carbon assimilation

To achieve the production of final products at a feasible scale, product yield and productivity must be considered under both light and dark conditions. The implementation of the sugar utilization pathway in cyanobacteria has successfully increased the product yield under either continuous or diurnal conditions.^10,11^ The heterologous expression of the galactose (GalP) or xylose (XylE) transporters, of xylose isomerase (XylA), and xylulokinase (XylB) from *E. coli* has resulted in the enhanced production of 2,3-butanediol (2,3-BDO), beside CO_2_ fixation, in cyanobacteria supplemented with glucose or xylose. Subsequently, metabolite profiling analysis was performed to assess the ratio of carbon assimilation from sugar and CO_2_ through the feeding of labeled [U-^13^C] glucose. Recently, the co-utilization of glucose and CO_2_ has been optimized to improve 2,3-BDO production and yield, by guiding the glucose flux into the central metabolism.^12^ Overexpression of key genes such as *zwf* (encoding for a glucose-6-phosphate dehydrogenase) and *gnd* (encoding for a 6-phosphogluconate dehydrogenase) in the oxidative pentose phosphate pathway, *prk* (encoding for a phosphoribulokinase), and *rbcLXS* (encoding for a ribulose-1,5-bisphosphate carboxylase/oxygenase (RuBisCo) subunit) led to the rewiring of glucose catabolism and the fixation of CO_2_, and increased the growth rate, glucose consumption, and 2,3-BDO production (1.1 g/L/d) in cyanobacteria. Photomixotrophic production using engineered cyanobacteria via the rewiring of metabolic pathways could be advantageous for high cell-density cultivation to increase the production yield, despite some concerns regarding the cost and the contamination of the sugar feedstock. In addition, D-lactic acid (2.17 g/L)^13^ and ethylene (821 ± 52 μL/L/h)^14^ have been photo-mixotrophically produced in engineered cyanobacteria using either acetate or xylose as additional carbon sources, respectively.

### Metabolic pathway rewiring to supply a key intermediate for improving production

**Figure 1.**
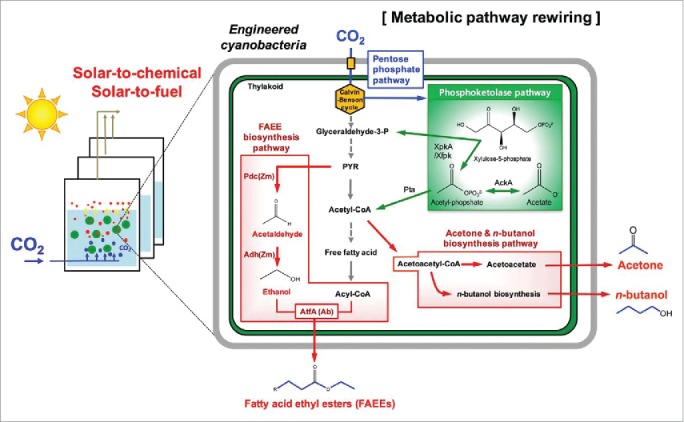
Development of engineered cyanobacteria through metabolic rewiring to construct solar-to-chemical and solar-to-fuel platforms. The rewiring of the heterologous phosphoketolase pathway to the pentose phosphate pathway in cyanobacteria has enhanced the levels of the acetyl-coA pool, resulting in an increased production of acetone,^18^ *n*-butanol,^19^ and fatty acid ethyl esters (FAEEs)^20^ from CO_2_. The phosphoketolase pathway is shown in the green box and the heterologous chemical-producing pathways are shown in red boxes. The carbon flux of CO_2_ is indicated by the blue arrow, and the carbon backbone that originated from CO_2_ is also shown in blue. XpkA/Xfpk, phosphoketolase; AckA, acetate kinase; Pta, phosphotransacetylase; Pdc (Zm), Pyruvate decarboxylase of *Zymomonas mobilis*; Adh (Zm), Aldehyde dehydrogenase of *Z. mobilis*.

Metabolic flux analysis^15^ and flux balance analysis^16^ have been performed in cyanobacteria to determine the carbon fluxes from CO_2_ and sugars, and to assist in the metabolic engineering of cyanobacteria. A relatively small fraction of carbon fluxes from CO_2_ were directed to fatty acids and the terpenoid biosynthesis pathway.^17^ Although the regulation of carbon partitioning in the cyanobacterial cell is not fully understood, it can be flexibly altered under certain conditions, such as nutrient deprivation and irradiance stress. Thus, it is necessary to perform pathway engineering in cyanobacteria to redirect carbon fluxes to the final product, in addition to reconstructing the metabolic pathway for the target product. Recent studies have addressed metabolic pathway rewiring to increase product yields by enhancing intermediate pools. For example, the engineered *Synechococcus elongatus* PCC 7942, a model cyanobacterium, harboring heterologous genes for acetone biosynthesis, did not produce any acetone from CO_2_ under light conditions.^18^ Subsequently, modular pathway engineering in *S. elongatus* PCC 7942 through the rewiring of the phosphoketolase (PHK) pathway to the acetone biosynthesis pathway has allowed the production of photosynthetic acetone from CO_2_ (Fig. 1). The rewired PHK pathway increased the level of the intermediate pool of acetyl-CoA that was used for improving acetone production. Consistently, the PHK pathway has been successfully rewired to the central metabolism of cyanobacteria to enhance the production levels of *n*-butanol^19^ and fatty acid ethyl esters,^20^ respectively. Another benefit of rewiring the PHK pathway is that the engineered cyanobacteria used for S2C and S2F platforms can be carbon efficient by bypassing pyruvate decarboxylation.^21,22^

### The plasticity of the cyanobacterial metabolism in the rewiring of metabolic pathways

The cyanobacterial metabolism is complex and plastic. This is supported by genetic and biochemical evidence that demonstrates the presence of 2-oxoglutarate decarboxylase and succinate semialdehyde dehydrogenase activities in the tricarboxylic acid cycle (TCA),^23^ the presence of the Entner-Doudoroff pathway,^24^ the glyoxylate cycle,^25^ and the gamma-aminobutyric acid shunt.^26^ Moreover, kinetic profiling of isotope-labeled metabolites has uncovered that the functional PHK pathway in *Synechocystis* sp. PCC 6803 is flexible, and has the potential to increase the efficiency of carbon metabolism and photosynthetic productivity.^27^ Independently, the engineering of cyanobacteria for the production of ethylene has revealed the plasticity of carbon metabolism by redirecting 37% of the fixed carbon flux into the TCA cycle, and by increasing the photosynthetic productivity for ethylene (718 ± 19 μL/L/h/OD_730_).^28^

Metabolic plasticity is also associated with the metabolic capabilities under various environmental growth conditions (e.g., photoautotrophic, photomixotrophic, or heterotrophic growth),^29,30^ and this has been demonstrated through the rewiring of metabolic pathways for the production of 2,3-BDO.^12^ Thus, the rewiring of metabolic pathways for directing carbon fluxes toward the desired products in cyanobacteria could facilitate the development of feasible S2C and S2F platforms.

## Conclusion

The status of solar-to-chemical and solar-to-fuel platforms for the production of value-added chemicals from CO_2_ has been addressed by focusing on the metabolic engineering of cyanobacteria. The rewiring of the metabolic pathways in cyanobacteria has allowed for the production of non-native chemicals, and facilitated carbon partitioning toward target chemicals using the S2C and S2F platforms. In addition, protein engineering^31^ and CRISPR-Cas9 genetic tools^32^ for metabolic engineering will likely promote the development of more efficient cyanobacterial cell factories. Moreover, photo-bioprocess engineering will be used for the generation of feasible S2C and S2F platforms.
